# Intensity-Modulated Radiotherapy (IMRT) following Conservative Surgery of the Supraglottic Region: Impact on Functional Outcomes

**DOI:** 10.3390/cancers14112600

**Published:** 2022-05-24

**Authors:** Daniela Alterio, Simona Marani, Valeria Zurlo, Stefano Filippo Zorzi, Annamaria Ferrari, Stefania Volpe, Francesco Bandi, Sabrina Vigorito, Maria Giulia Vincini, Sara Gandini, Aurora Gaeta, Cristiana Iuliana Fodor, Alessia Casbarra, Mattia Zaffaroni, Anna Starzyńska, Liliana Belgioia, Mohssen Ansarin, Cynthia Aristei, Barbara Alicja Jereczek-Fossa

**Affiliations:** 1 Division of Radiation Oncology, European Institute of Oncology (IEO), Istituto di Ricovero e Cura a Carattere Scientifico (IRCCS), 20141 Milan, Italy; daniela.alterio@ieo.it (D.A.); maranisimona@libero.it (S.M.); annamaria.ferrari@ieo.it (A.F.); stefania.volpe@ieo.it (S.V.); mariagiulia.vincini@ieo.it (M.G.V.); cristiana.fodor@ieo.it (C.I.F.); alessia.casbarra@ieo.it (A.C.); mattia.zaffaroni@ieo.it (M.Z.); barbara.jereczek@ieo.it (B.A.J.-F.); 2 Department of Otolaryngology and Head and Neck Surgery, European Institute of Oncology (IEO), Istituto di Ricovero e Cura a Carattere Scientifico (IRCCS), 20141 Milan, Italy; stefano.zorzi@ieo.it (S.F.Z.); francesco.bandi@ieo.it (F.B.); mohssen.ansarin@ieo.it (M.A.); 3 Department of Oncology and Hemato-Oncology, University of Milan, 20122 Milan, Italy; 4 Unit of Medical Physics, European Institute of Oncology (IEO), Istituto di Ricovero e Cura a Carattere Scientifico (IRCCS), 20141 Milan, Italy; sabrina.vigorito@ieo.it; 5 Department of Experimental Oncology, European Institute of Oncology (IEO), Istituto di Ricovero e Cura a Carattere Scientifico (IRCCS), 20141 Milan, Italy; sara.gandini@ieo.it (S.G.); aurora.gaeta@ieo.it (A.G.); 6 Department of Oral Surgery, Medical University of Gdańsk, 80-210 Gdańsk, Poland; anna.starzynska@gumed.edu.pl; 7 Radiation Oncology Department, Istituto di Ricovero e Cura a Carattere Scientifico (IRCCS), Ospedale Policlinico San Martino, 16132 Genova, Italy; liliana.belgioia@unige.it; 8 Health Science Department (DISSAL), University of Genoa, 16132 Genova, Italy; 9 Radiation Oncology Section, Department of Medicine and Surgery, University of Perugia and Perugia General Hospital, 06123 Perugia, Italy; cynthia.aristei@unipg.it

**Keywords:** intensity-modulated radiotherapy, toxicity, supraglottic region, organ preservation

## Abstract

**Simple Summary:**

The present study has been suggested by the previous experience of our group showing that patients treated with conventional radiotherapy (named 3D conformal radiotherapy—3D-CRT) performed after conservative surgery (CS) for tumors of the supraglottic regions experienced a high rate of severe long-term toxicity. Therefore, we reported the toxicity profile of a similar cohort of patients treated with a high-precision radiotherapy technique (named intensity-modulated radiotherapy—IMRT). Moreover, to investigate the advantage of IMRT, we performed a comparison with a historical cohort treated with 3D-CRT. Results showed that patients treated with IMRT achieved a very low toxicity profile and comparison with 3D-CRT was in favor of IMRTs. Therefore, we believe that the results of the present study provide preliminary findings on the potential of IMRT in improving the toxicity profile of patients treated with surgical organ preservation strategies for laryngeal tumors.

**Abstract:**

The aim of the present study was to investigate the role of intensity-modulated radiotherapy (IMRT) on the toxicity profile of patients treated with conservative surgery (CS) of the supraglottic (SG) region. Data on patients treated with CS and postoperative radiotherapy (PORT)-IMRT were prospectively collected. Results. In total, 20 patients were analyzed. Of these, six patients (35%) required the positioning of a temporary tracheostomy. The functional larynx preservation rate was 95%. Females had a higher risk of both endoscopic intervention and chondronecrosis, while the median age was significantly higher in patients requiring enteral nutrition. The incidence of long-term severe toxicities was lower in patients treated with IMRT than in the historical 3D-CRT cohort. Patients who had received PORT-IMRT achieved a lower rate of permanent laryngeal and swallowing dysfunctions. Overall, results from the comparison with the historical 3D-CRT cohort favor the IMRTs.

## 1. Introduction

Organ preservation for locally advanced laryngeal tumors includes either conservative surgery (CS) (+/− adjuvant treatment) or chemoradiation [1,2,3,4]. Studies on indirect comparison between these strategies have shown comparable oncological outcomes [1,2,5]. Therefore, in daily clinical practice, the choice depends on the individual surgeon’s experience, each center’s equipment, and each patient’s preference.

While the outcomes are similar, the combination of CS and postoperative radiotherapy (PORT) can be burdened by a significant rate of relevant long-term side effects, especially when compared to the non-surgical strategy [6,7,8]. In particular, the treatment of the supraglottic (SG) region is associated with a higher rate of dysphagia and respiratory dysfunction, due to the crucial role of this area in the swallowing and breathing processes [9]. Consistently, up to one-third of patients treated with PORT following SG laryngectomy may require enteral nutrition and/or permanent tracheostomy, or experience chondronecrosis [10,11,12]. Of note, considering PORT, most literature data are based on treatments delivered in the early 2000s, when the 3D conformal radiotherapy (3D-CRT) technique was the standard of care.

Intensity-modulated radiotherapy (IMRT) has been associated with a reduced toxicity profile in several head and neck tumors both in the curative and postoperative settings. However, its impact in SG cancer is currently supported by limited data [13,14,15,16]. The introduction of IMRT, as well as the addition of concurrent chemotherapy, has been associated with improved oncological outcomes in Dutch patients treated with chemoradiation for SG tumors [17]. These data suggest that the reduction of long-term laryngeal toxicity allowed by IMRT, might have an impact on the patients’ prognosis by minimizing treatment-related fatal events. Nevertheless, whether radiation technological advances could improve early and late radiation-related side effects in the adjuvant treatment of SG cancers has not yet been explored.

Therefore, to analyze the impact of IMRT on the toxicity profile of patients treated with a larynx-preservation strategy, we reported outcomes of prospectively enrolled consecutive patients treated at our Institute with CS involving the SG region followed by PORT-IMRT for locally advanced laryngeal cancers. Additionally, to fully investigate the putative advantage of IMRT over a 3D conformal technique, we performed and described a clinical comparison between patients treated with PORT-IMRT and a historical cohort of patients treated with PORT-3D CRT.

## 2. Materials and Methods

### 2.1. Patients Treated with CS and PORT-IMRT

Data on patients treated at the European Institute of Oncology IRCCS, Milan, Italy, with CS of the SG region and PORT from 2013 to 2019 were prospectively collected. Inclusion criteria were locally advanced carcinoma (stage III and IV according to the AJCC 7th Edition) treated with CS and PORT-IMRT performed at our Institute and a minimum follow-up of 6 months. Chemoradiation was also allowed, while previous surgery or RT in the head and neck region, non-squamous histologies, and treatment with surgery alone or eligibility for palliative treatments represented the exclusion criteria. Additionally, the availability of written informed consent for the anonymized use of data for clinical research purposes was verified for each enrolled patient. The study was notified to and approved by the Institutional Ethical Committee (RTP R044).

Locoregional staging was assessed by physical examination (fibroendoscopy performed on all patients), radiological imaging including computed tomography (CT), and, in selected cases, magnetic resonance imaging (MRI) and/or ultrasonography (US). The presence of distant metastases was excluded by a total-body CT scan and/or fluoro-deoxy-glucose positron emission tomography (FDG-PET).

The treatment strategy (chemoradiotherapy versus CS +/− adjuvant radiotherapy) was defined within the institutional Head and Neck Tumor Board, whose discussants considered tumor extension, expected functional results, and patients’ clinical characteristics (e.g., age, comorbidities, and preference).

Following multidisciplinary team discussion, indication for a surgical organ preservation strategy was given in the case of T1–T3 and selected T4 primary tumors of the larynx according to international and institutional guidelines. Surgery was performed both with open-neck or endoscopic procedures with either a CO_2_ laser or a robot-assisted technique. Specifically, endoscopic surgery was proposed in case of early-stage tumors (T1–T2 and T3 with limited involvement of the pre-epiglottis space) cN0–N2c, good laryngeal exposure, and no cardiopulmonary comorbidity. Open-neck CS was preferred either for early-stage tumors (T1–T3) with a contraindication to the endoscopic approach, or in case of clinically positive lymph nodes at diagnosis (N1–N2c). A more extensive surgery including the base of the tongue and/or the glottic region and/or the arytenoids and pyriform sinus was performed in case of mucosal involvement of the base of the tongue and/or the pyriform sinus and/or the glottis and/or in case of impairment of arytenoid mobility. Indications for the removal of one arytenoid cartilage, mono- or bilateral neck nodal dissection, and the extent of the surgical procedure were based on clinical and radiological staging.

Indication for PORT was given for patients with pT3 (in case of close/positive surgical margins and/or perineural invasion) and pT4 and/or close/positive surgical margins and/or ≥2 positive lymph nodes and/or lymph node extracapsular extension and/or adverse biological characteristics (perineural invasion, lymph vascular infiltration, and grading). IMRT was performed via a volumetric modulated arc therapy (VMAT) technique. The total prescription total doses were as follows: 66 Gy (2 Gy/fraction) for lymph node(s) with extracapsular extension (high dose—HD-volume), 59.4 Gy (1.8 Gy/fraction) for the primary tumor bed (pT3, pT4) or pathologic lymph nodes without extracapsular extension (high risk—HR-volume), and 56.1 (1.7 Gy/fraction) and prophylactic irradiation of neck lymph nodes (low risk—LR-volume). In case of positive surgical margins, the prescription doses ranged from 60 to 66 Gy. A simultaneous integrated boost (SIB) technique was used for all patients. The remnant larynx was contoured in all patients, and the dose distribution was optimized to avoid hotspot areas (>107% of the prescribed dose). Platinum-based concomitant chemoradiotherapy was proposed for “high-risk” patients (presence of positive surgical margins or extracapsular spread).

After the completion of PORT, follow-up consultations were planned every 3 months for the first 2 years, every 4 months for the subsequent 3 years, and every 6 months thereafter. Chest X-rays were required once a year. Fibroendoscopy was performed at every clinical examination. Radiological examinations (US, CT, MRI, and FDG-PET) were also required periodically. The dosage of thyroid hormone was assessed every 6 months.

Local-regional relapse was defined as the re-appearance of cancer in the site of the primary tumor and/or neck lymph nodes at any time after the end of RT.

Data on the acute and late toxicity profiles were retrieved. Acute toxicity was considered as any toxicity that occurred during 6 months from the end of the RT course. Late toxicity was considered the worst toxicity event that occurred from 6 months after RT ended to the last follow-up. Acute and late radiation-related side effects were evaluated according to the Radiation Therapy Oncology Group (RTOG)/European Organization of Research and Treatment of Cancer (EORTC) scoring system (EORTC-RTOG) (for anatomic mucositis, skin toxicity, and laryngeal edema) and the Common Terminology Criteria Adverse Event (CTCAE V4.03), for all other toxicities. Pain intensity was evaluated using a Numeric Pain Rating Scale (NRS) [18,19]. Laryngeal stenosis (whether requiring surgical intervention or not), chondronecrosis, and temporary or permanent tracheostomy were recorded. Defective swallowing (defined based on the need for enteral nutrition) and the presence of definitive percutaneous gastrostomy (PEG) were also reported.

Data on functional outcomes and patients’ quality of life (QoL) were also collected. Swallowing was evaluated by the Penetration–Aspiration scale (PAS—graded from 1/no symptoms to 8/maximum aspiration), while the quality of voice was collected by the Voice Handicap Index (VHI) questionnaire (graded as 0/never symptoms to 4/always) considered for physics and functional and emotional areas. The Quality-of-Life European Organisation for Research and Treatment of Cancer (EORTC QLQ—H&N35) questionnaire was completed for alive patients at the last follow-up. Questions from 32 to 60 refer to patients’ distress due to swallowing dysfunction, with possible answers ranging from 0 (no distress) to 3 (maximum distress). Questions from 61 to 65 refer to objective symptoms (requirement of pain medications, dietary supplements, nasogastric tube, and weight loss), with allowed answers being either 0 (no) or 1 (yes).

Outcome analyses were performed, as well: overall survival (OS) was calculated as the time (days) from the end of treatment to the last contact at follow-up or death; local progression-free survival (L-PFS) was calculated as the days between the end of treatment and local relapse or death. Finally, disease-free survival (DFS) was retrieved considering the days between the end of PORT and the first event among local relapse, metastatic progression, and death.

### 2.2. Comparison with an Historical Cohort of Patients Treated with the 3D Conformal Technique

Data on 32 patients with SG tumors treated with CS and postoperative 3D CRT had previously been reported by our group [12] and were used as a comparator to the present cohort for the following severe long-term toxicity: stenosis and/or edema requiring endoscopic dilation, enteral nutrition (percutaneous gastrostomy PEG) or tracheostomy at last follow-up, and chondronecrosis. Risk factors for the development of the above-listed side effects were gender, age, radiation treatment technique (IMRT vs. 3D conformal), surgical approach (endoscopic vs. open surgery), surgical extension (removal of base of tongue or one arytenoid), arytenoid removal (yes vs. no), and presence of tracheostomy during the radiation course. Categorical variables were summarized with frequencies and percentages while, for continuous variables, median, minimum/maximum, or interquartile range were reported. Differences between treatment groups (3D and IMRT) were tested using Pearson’s chi-square test or Fisher’s test for categorical variables and Wilcoxon rank-sum test for continuous variables.

## 3. Results

### 3.1. Patients Treated with CS and PORT-IMRT

Among the 68 patients with locally advanced SG cancers treated at our institute with CS in the considered period, 20 matched the inclusion criteria and were included in the analysis. Sixteen (80%) patients were men, and the median age was 60 (IQR; 57.5–64.4) years. Moreover, 6 and 13 patients were active and former smokers, respectively, while one patient had never smoked. Most patients (60%) had at least one relevant comorbidity. In all, 9 (45%) patients had pathological stage III, and 11 (55%) had stage IV (IVa and IVb in 8 and 3 patients, respectively), according to the AJCC TNM 7th Edition [20]. Pathologic stages according to primary tumor (T) and lymph node (N) involvement are presented in Table 1.

Perineural (PNI) and lymphovascular (LVI) invasion were found in 5 and 3 patients, respectively, while 10 had grade 3 cancers. Extracapsular extension was reported for seven patients. Surgical procedures and the status of resection margins are reported in Table 2.

One arytenoid was completely and partially removed in six and two patients, respectively. Out of the 12 patients who underwent a surgical procedure extended to the pyriform sinus or base of tongue, 4 had one arytenoid removed. All six patients with positive/close surgical margins received open surgery. Of these, one and five patients had T2 and T3 stage, respectively. In three cases, the tracheostomy was precautionarily left in place during the PORT-IMRT course. The median interval between surgery and PORT was 60 days (IQR 54–69 days).

Five patients received adjuvant chemoradiation. Median PORT duration was 44 days (interquartile range—IQR: 44–49 days). All patients completed the planned RT treatment. The total dose prescription for the HD, HR, and LR volumes were 66 (IQR 63–66 Gy), 59.4 (IQR 59.4–60), and 56.1 (IQR 54–56.1) Gy, respectively. The median dose prescription to the remnant larynx was 60 Gy (IQR 0–60 Gy). The median total dose prescription to the remnant larynx in case of positive surgical margins (six patients) was 60 (range 59.4–66) Gy.

Median follow-up was 75 (IQR, 58–115) months. Five patients died, two due to tumor progression and three due to non-cancer related causes (namely, respiratory distress, metastases from prostate cancer, and unknown cause occurring after the onset of central nervous symptoms). Two patients experienced distant metastatic progression, while no locoregional recurrences were registered during the follow-up for the whole population.

Overall, none of the considered oncological outcomes (OS, L-PFS and DFS) were significantly different between the 3D-CRT and IMRT subgroups.

#### 3.1.1. Acute Toxicity

In 18 (90%) patients, a temporary tracheotomy was positioned during the surgical procedure (1 and 17 patients treated with endoscopic and open surgery procedures, respectively) and was removed after a median time of 14 days. All but one patient had no major surgery-related complications. One patient required a surgical second look due to a neck hematoma. During IMRT-PORT, three patients maintained their tracheostomy in place (due to postsurgical chondritis and laryngeal function impairment) while no patient required enteral nutrition. Details on the acute toxicity profile have been reported in Supplementary Materials (Table S1). One patient developed acute dyspnea due to severe laryngeal edema three months after the end of RT and required a tracheostomy, which was left in place for 28 days.

#### 3.1.2. Late Toxicity

At one year from the end of PORT, toxicity data were available for 18 patients, while the toxicity profile at the last follow-up was collected for 19 patients (Table 3).

In the long term, six patients (35%) required the positioning of a temporary tracheostomy:

The tracheostomy was left in place in five patients for a median period of 23 days (IQR 19–90 days).

Two patients required temporary enteral nutrition (percutaneous gastrostomy and nasogastric tube) positioned after 64 and 4 months from the end of RT, respectively. Both patients also had respiratory distress requiring tracheostomy. Finally, at the last follow-up, all but one patient were free from both tracheostomy and enteral nutrition, and the functional larynx preservation crude rate was 95%.

A detailed description of the patient, treatment, and tumor characteristics, as well as the absorbed dose to the remnant larynx and the toxicity profile, is presented in the Supplementary Materials (Table S2).

Overall, among the eight patients who underwent arytenoid removal, only one developed a laryngeal stenosis, requiring temporary tracheostomy, while no laryngeal dysfunction occurred for the remnant patients. Of the two patients who had the tracheotomy in place during the RT course, one patient developed laryngeal stenosis requiring temporary tracheostomy.

Of the 17 patients treated with an open partial laryngectomy, six (35%) needed temporary tracheostomy during their follow-up: most of them (five of six) were treated with a more extensive surgery (two patients had arytenoid removal, three had surgical procedure extended to base of tongue and/or pyriform sinus). Of the three patients who underwent endoscopic surgery, one patient required both PEG and tracheostomy.

#### 3.1.3. Functional Outcomes

PAS (ranging from 1 to 8) was evaluated in 16 patients at baseline with a median value of 2 (mean 3, range 1–7). At last follow-up, PAS was available for 15 patients. After a median follow-up of three years, the median value was 4 (IQR 1–8). For 11 patients, the PAS evaluation was performed both at baseline and at the last follow-up, resulting in stable, better, and worse values for four (36%), three (28%), and four (36%) patients, respectively.

The EORTC QLQ—H&N35 questionnaire was completed by 17 patients. A mean and median value of 7 (range 0–26) was recorded for questions 32–60. For questions 61–65, the mean and median value was 1 (range 0–3) with six (35%) patients achieving a total score of 0 (no discomfort at all).

The results of the VHI questionnaire were evaluated according to the physical (range 0–36), functional (range 0–40), and emotional areas (range 0–40). A mean value of 13 was detected. For physical symptoms, the mean and median values were 16 (range 0–27) and 11 (range 0–31), respectively. For the emotional area, the mean and median values were 8 and 5 (range 0–35).

### 3.2. Comparison with an Historical Cohort

In all, 52 patients (32 patients represented the historical cohort treated with 3D CRT and 20 patients were treated with IMRT) represented the whole cohort of analyzed patients.

The two cohorts were homogenous as per gender, age, tumor grading, surgical margins, extracapsular extension, primary tumor stage, and pathologic stage, while nodal involvement was significantly lower in patients treated with IMRT (65.0% vs. 84.4%, *p* = 0.01).

Treatment characteristics of the IMRT and 3D CRT cohorts were also comparable for the presence of tracheostomy during RT (15% vs. 37.5%, respectively, *p* = 0.12), enlarged surgical procedure (15% vs. 6%, respectively, *p* = 0.36), and arytenoid removal (40% vs. 15%, respectively, *p* = 0.10). Only open partial laryngectomy was significantly more frequent among patients treated with IMRT compared to the historical cohort (85% vs. 40%, respectively, *p* = 0.002).

The incidence of long-term severe toxicity in patients treated with 3D CRT and IMRT was as follows: persistent aspiration/pneumonia 6% vs. 0%, acute respiratory distress 3% vs. 15%, laryngeal stenosis requiring endoscopic dilatation 21% vs. 15%, laryngeal necrosis 6% vs. 0%, and permanent tracheostomy 12.5 vs. 5%, respectively. None of the patients required permanent gastrostomy in both groups.

Despite the higher number of late toxicity events in the historical cohort compared to that in patients treated with IMRT, none of the differences reached statistical significance. Female gender was statistically associated with a higher incidence of temporary tracheostomy after RT (50% of female vs. 16% male, *p*-value = 0.05). Females also experience a statistically significant higher incidence of chondronecrosis (*p* = 0.02).

The surgical approach (open vs. endoscopic) and arytenoid removal showed a trend toward a higher risk (*p* = 0.09) of temporary and permanent tracheostomy, respectively.

Median age was significantly higher in those with enteral nutrition during the follow-up (median age 70 vs. 60 years, *p*-value = 0.05). Details of these correlations are reported in the Supplementary Materials (Table S3).

## 4. Discussion

The management of locally advanced laryngeal cancers involving the SG region with a surgical organ-preservation approach is challenging because of the high risk of treatment-related functional impairment of the remnant larynx [21]. Consensus guidelines suggest proposing CS to patients who are not expected to require PORT, in order to avoid the long-term toxicity related to the combined treatment [6,7,22]. However, literature data report that PORT is required in about two-third of patients with variable incidence of severe long-term side effects [23]. Results of the present analysis show that CS followed by PORT-IMRT warrants a favorable acute and long-term toxicity profile, with a low rate of permanent laryngeal dysfunction.

### 4.1. Toxicity Profile of CS Followed by PORT

Data on the toxicity profile of patients treated with CS and PORT-IMRT are lacking. The majority of previously published series referred to patients treated in the pre-IMRT era (2D or 3D conformal technique) [10,11,24,25]. Steniger et al. reported a rate of permanent gastrostomy, persistent aspiration, and permanent tracheostomy of 35%, 30%, and 23%, respectively, in 17 patients treated with 3D PORT (Steniger). Moreover, acute respiratory distress occurred in about one-third of the patients. Garibaldi et al. reported no patients requiring permanent tracheostomy, while 3% of patients suffered from acute respiratory distress (Garibaldi) (Supplementary Materials Table S4). Considering literature data, the current series showed a favorable toxicity profile both for swallowing dysfunctions (0% permanent gastrostomy, aspiration) and permanent laryngeal impairment (5% permanent tracheostomy with 0% cartilage necrosis). Nevertheless, laryngeal-related toxicity with transient dysfunction (acute respiratory distress and laryngeal stenosis requiring endoscopic dilatation) remains a critical aspect that needs to be monitored during the follow-up period.

### 4.2. Risk Factors for Radiation-Related Long-Term Toxicity

In the present series, female gender and older age were found to be factors correlated with a higher risk of developing long-term side effects in terms of laryngeal stenosis and enteral nutrition, respectively. This is in line with the general knowledge that the smaller laryngeal volume exposes women to a higher risk of laryngeal impairment regardless of the treatment approach and that older patients could have more difficulties in following rehabilitation programs required after conservative surgery of the larynx.

#### 4.2.1. Surgical Procedures

The surgical approach (endoscopic versus open partial laryngectomy) could also have an impact on the overall toxicity profile with endoscopic procedures characterized by more efficient postsurgical recovery [26,27,28,29]. Therefore, findings of the present cohort are in line with the literature data since the open surgery approach showed a trend toward a higher risk for temporary tracheostomy.

The presence of a tracheostomy during PORT has been traditionally considered to increase the risk of upper-airway obstruction by favoring laryngeal or tracheal chondritis [11]. In contrast, other authors advise that postsurgical tracheostomy could be left in place to prevent possible sequelae, which could also worsen in to fatal complications [24,30]. Laccourreye et al. found that 3.3% of patients died due to treatment-related toxicity, and the authors hypothesize that an early intervention with total laryngectomy could have prevented the occurrence of such events [24]. In the present cohort, 90% of patients did not have a prophylactic tracheostomy during and after PORT, and this could have favored the low rate of permanent laryngeal dysfunction. On the other hand, this finding could justify the high-rate transient dysfunction compared to both our historical data and published series in which the rate of tracheostomy left in place during PORT could be higher. Therefore, these data suggest that the use of prophylactic airway protection should be evaluated on a case-to-case basis according to the risk of developing severe, although transient, laryngeal dysfunction.

#### 4.2.2. Total Dose to the Remnant Larynx

Treated volumes (first-level lymph nodes vs. total neck irradiation) and total dose >50 Gy to the remnant larynx were found to be correlated with the probability of developing grade 2+ laryngeal toxicity in a series of 56 patients [10]. The multivariate analysis confirmed that total dose was the only independent factor. In the present cohort, the maximum and mean dose to the remnant larynx was not different between patients who required temporary tracheostomy and those who did not. This result could be explained both by the small sample size of our IMRT cohort and by the fact that the majority of our patients (70%) received a median dose of 60 Gy to the tumor surgical bed.

The main determinant of the radiation dose to the surgical tumor bed is represented by the pathologic margin. Notwithstanding the aim of surgery to achieve a microscopically radical resection, positive margins could occur in up to one-third of patients treated with CS for SG cancers [9]. Radiation oncologists recommended a dose of 62–66 Gy in case of positive margins for the majority of head and neck cancers, but the optimal dose to be administered to the remnant larynx in case of close/positive margins after a CS approach has not been standardized yet. Indeed, some authors speculate that the putative low tumor burden of the residual microscopic disease due to the laryngeal peculiar anatomy with cartilage barriers, might require a lower dose to achieve adequate local control [10]. In line with this concept, some institutions currently limit the dose to the larynx to 55 Gy after a horizontal supraglottic laryngectomy [31]. Moreover, Fiz et al. reported that patients with multiple deep positive margins could have a higher risk of recurrence compared to those with one superficial positive margin in early-stage glottic tumors [32]. In line with this approach, Carta et al. considered surgical margins as positive only if the tumor was present at the level of either one deep resection margin or more than one positive superficial margin [33]. In the present cohort, 60% of patients had locally advanced tumors, and 30% had close/positive surgical margins. Our analysis did not allow definitive conclusions to be drawn on the association between the risk of laryngeal impairments and the absorbed dose. Therefore, although according to the literature data it seems to be reasonable to prescribe a total dose of 50–56 Gy in case of locally advanced stage tumors (pT3–pT4), whether a dose <60 Gy could reduce the toxicity profile without jeopardizing oncologic results in case of close/positive margins warrants further investigations.

### 4.3. Functional Outcomes

Data on QoL and functional outcomes have been rarely reported in surgical series of patients treated for SG cancers. Ambrosh et al. compared patients treated with CS only with those submitted to PORT [4]. Results showed worse outcomes in patients treated with a combined approach, with a median MDADI composite score of 70 and 90 among irradiated and non-irradiated patients, respectively. Similarly, the median VHI score was higher in those who had received PORT (49 vs. 16, respectively). Comparatively, our patients experienced a median VHI score of 13. On the contrary, the pre- and post-PORT PAS scale was either stable or improved in about two-third of patients in our series. Results of the QoL questionnaire showed a very low rate of discomfort for the majority of the analyzed items. In conclusion, the functional outcomes of patients treated with IMRT seems to be quite favorable. Considering the paramount importance of voice and swallowing evaluation as well as the patients’ QoL, the prospective collection of such data is recommended.

### 4.4. Advantages of IMRT over 3D CRT

Several authors recommend the optimization of dose distribution by the use of more conformal radiation techniques in order to optimize the absorbed dose to the remnant larynx [3,4,24,25].

In locally advanced supraglottic tumors, a laryngeal-preservation approach performed through a conservative surgery has been suggested to achieve higher loco-regional control (FFR) with comparable 5-year overall survival (OS) compared to chemoradiation [1,2,5]. In line with the literature data, the experience of our center showed an OS and locoregional control of 96% and 67%, respectively [34].

**Figure 1:**
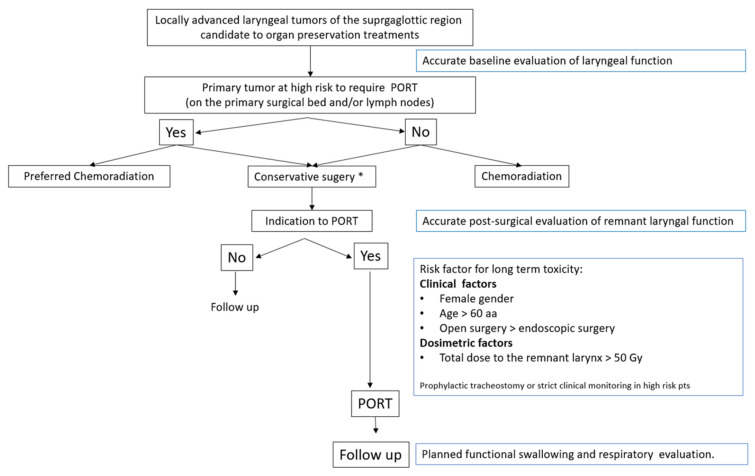
Workflow for patients with locally advanced laryngeal cancers involving the supraglottic region who are candidates for an organ-preservation approach. * High-volume centers with expert surgeons and availability of multidisciplinary rehabilitation service.

According to literature data and results of the present analysis, some suggestions could be provided for daily clinical practice. First, an accurate baseline evaluation of the laryngeal function should be performed, regardless of the treatment strategy of choice, for any SG tumor candidate for preservation. Moreover, considering the high risk of functional laryngeal impairment in patients receiving a combined approach (conservative surgery followed by PORT), a surgical approach should be proposed only in high-volume institutions equipped with a multidisciplinary rehabilitation service. Furthermore, when PORT is indicated, a postsurgical functional evaluation of the remnant larynx is highly recommended in order to quantify swallowing defects, if any, and to begin rehabilitation exercises, especially considering clinical factors (namely, female gender, age >60 years). Figure 1 presents a workflow of the main elements to consider for the optimization of tumors involving the SG region in the setting of a surgical organ-preservation strategy.

We are aware that the main pitfall of the present work is represented by the limited number of analyzed patients. Moreover, despite the more favorable toxicity profile, comparison with a historical cohort of patients treated with 3D CRT showed that patients treated with IMRT did not experience a statistically significantly lower number of side effects. This finding might be explained by the fact that confounder factors (higher rate of open surgery among patients treated with IMRT) could have underestimated the clinical advantage of a more conformed technique. Therefore, whether the reduction of absorbed dose to these structures could improve clinical outcomes is yet to be proven. Despite these weaknesses, to the best of our knowledge, this study reports a full analysis (objective toxicities and QoL parameters) of patients treated with IMRT in the setting of conservative surgery of the SG larynx providing high-quality data (homogeneity of both surgical procedures and radiation technique) provided by a high-volume center. Therefore, due to the paucity of literature data on the topic, we believe that our work provides preliminary findings on the potential of IMRT in improving the functional outcomes of SG cancers patients treated with a CS approach. Moreover, we highlight a multidisciplinary set of useful information for daily clinical practice and encourage further studies on larger cohorts aiming at optimizing clinical management and refining treatment strategies within the surgical organ-preservation approach.

## 5. Conclusions

The results of our study showed that patients treated with IMRT after CS for SG achieved a low rate of permanent laryngeal and swallowing dysfunctions. Both the literature data and the present analysis strongly suggest that the toxicity profile depends on several factors such as patients’ characteristics, surgical approach, and radiation doses. Further investigations on larger cohorts with longer follow-up are needed to confirm these encouraging preliminary findings.

## Figures and Tables

**Table 1:** Pathological tumor and nodal staging.

	pT1	pT2	pT3	pT4a	Total
pN0	0 (NE) *	0 (NE)	5	2	7
pN1	1	1	1	0	3
pN2a	0	1	1	0	2
pN2b	1	0	1	0	2
pN2c	0	1	3	0	4
pN3	1	1	0	0	2
Total	3	4	11	2	20

* Not eligible.

**Table 2:** Treatment characteristics.

Surgery Characteristics			Number of Patients (*n* = 20, %)
Surgical approach	Endoscopic		3 (15)
	Robot-assisted	1	
	CO_2_-laser	2	
	Open surgery		17 (75)
	Supracricoid laryngectomy (OPHL I)	10	
	Supraglottic laryngectomy (OPHL II)	7	
Removal of one arytenoid	Yes		8 (40)
	No		12 (60)
Lymph node dissection	Monolateral		6 (30)
	Bilateral		12 (60)
	No		2 (10)
Extend of surgical procedure	To pyriform sinuses (mucosa)		8 (40)
	To tongue base (mucosa)		4 (20)
	No		8 (40)
Surgical margins	Positive		3 (25)
	Close (<5 mm)		3 (25)
	Negative		14 (50)

Abbreviations: OPHL = open partial horizontal laryngectomy.

**Table 3:** Toxicity profile at one year from the end of radiotherapy.

Toxicity	G0 (%)	G1 (%)	G2 (%)	G3 (%)
At 1 year (18 patients)				
Anatomical laryngeal edema	4 (22)	9 (50)	4 (22)	1 (6)
Functional laryngeal edema	12 (67)	5 (28)	0	1 (6)
Dysphagia	10 (55)	7 (39)	1 (6)	0
Xerostomia	5 (28)	13 (72)	0	0
At last follow-up >1 year (19 patients)				
Anatomical laryngeal edema	12 (63)	2 (10)	4 (21)	1 (5)
Functional laryngeal edema	13 (68)	4 (21)	1 (5)	1 (5)
Dysphagia	12 (63)	6 (31)	1 (5)	0
Xerostomia	5 (26)	14 (74)	0	0

## Data Availability

Data is contained within the article and supplementary material.

## Supplementary Materials

The following supporting information can be downloaded at https://www.mdpi.com/article/10.3390/cancers14112600/s1, Table S1: Acute toxicity profile of 20 patients treated with CS and PORT IMRT for SG locally advanced cancers according to Common Terminology Criteria Adverse Event (CTCAE) scale (v. 4.03). Table S2: Detailed description of the 20 patients treated with conservative surgery and postoperative radiotherapy for supraglottic tumors. Table S3: Correlation between risk factors and long-term side effects. Table S4: Toxicity profile of patients treated with conservative surgery and postoperative radiotherapy for SG performed with no-IMRT techniques and current series.

## Abbreviations

CS	Conservative surgery
CT	Computed tomography
CTV	Clinical target volume
CTV HD	Clinical target volume “high dose”
CTV HR	Clinical target volume “high risk”
CTV LR	Clinical target volume “low risk”
Dmax	Maximum dose
Dmean	Mean dose
FDG-PET	Fluoro-deoxy-glucose positron emission tomography
FSUs	Functional swallowing units
FOM	Floor of the mouth
GGS	Genioglossus muscle
GTV	Gross tumor volume
HLE	Elevation of the hypolarynx
HN-SCC	Laryngeal squamous cell carcinomas
HSG	Hyoglossus/styloglossus muscle complex
IMRT	Intensity-modulated radiotherapy
ITM	Intrinsic muscles of the tongue
LFS	Laryngectomy-free survival
LPM	Longitudinal pharyngeal muscles
LVI	Lymph vascular infiltration
MRI	Magnetic resonance imaging
OAR	Organ at risk
OS	Overall survival
PAS	Penetration–Aspiration Scale
PDS	Posterior digastric/stylohyoid muscle complex
PL	Partial laryngectomy
PEG	Percutaneous endoscopic gastrostomy
PNI	Perineural invasion
PORT	Post-operative radiotherapy
PTV	Planning target volume
QoL	Quality of life
RT	Radiotherapy
S	Surgery
SCL	Supracricoid laryngectomy
SL	Supraglottic laryngectomy
SDI	Sociodemographic Index
TBR	Tongue base retraction
THM	Thyroid muscle
US	Ultrasonography
VHI	Voice Handicap Index
VMAT	Volumetric modulated arc therapy
