# Supplementary material for: Intensity-Modulated Radiotherapy (IMRT) following Conservative Surgery of the Supraglottic Region: Impact on Functional Outcomes
Source: Cancers (Basel). 2022 May 24;14(11):2600. doi: 10.3390/cancers14112600 (PMC9179846; doi:10.3390/cancers14112600)
Supplement: Supplementary file 1 [file cancers-14-02600-s001.zip › cancers-1667765-supplementary.pdf]

cancers

Supplementary Materials

Table S1. Acute toxicity profile of 20 patients treated with CS and PORT IMRT for SG locally advanced cancers according Common Terminology Criteria Adverse Event (CTCAE) scale (v 4.03)

Toxicity	Grade		
	G0 (%)	G1 (%)	G2 (%)
Anatomic mucositis *	0	17 (85)	3 (15)
Functional mucositis	2 (10)	15 (75)	3 (15)
Dysphagia	2 (10)	14 (70)	4 (20)
Xerostomia	3 (15)	14 (70)	3 (15)
Skin toxicity *	0	11 (55)	9 (45)
Dysphonia	2 (10)	11 (55)	4 (20)
Weigh loss	15 (75)	5 (25)	0

*Assessed per the Radiation Therapy Oncology Group (RTOG)/European Organization of Research and Treatment of Cancer (EORTC) scoring system (EORTC-RTOG). Median Mean weight loss was 1.45 Kg (IQR 3.35 -0.45 Kg). The median value of maximum pain was 5 (range 3-5).

Table S2. Detailed description of the 20 patients treated with conservative surgery and postoperative radiotherapy for supraglottic tumors

Gender	Age	Smoking habit	Comorbidities	Surgery	Enlarged surgery	Arytenoid removal	Tumor grading	Surgical margins	Perineural invayeson	Lymphovascular invayeson	Extracapsular extenyeson	Pathologic tumor stage	Pathologic Lymph node stage	Concurrent chemotherapy	Tracheostomy during radiotherapy	Mean dose absorbed to the remnant larynx	Maximum dose absorbed to the remanant	Temporary tracheostomy during follow-up	Tracheostomy at last follow-up	Enteral nutrition during follow-up	Enteral nutrition during follow-up
M	74	Ex	Yes	E	No	No	G3	Neg	No	No	Yes	T1	N1	No	No	60.77	67.29	Yes	Yes	Yes	Yes
M	58	Ex	No	O	Yes	No	G3	Close	Yes	Yes	Yes	T2	N3	Yes	No	62.59	64.35	No	No	No	No
M	70	No	No	O	No	Yes	G1	Pos	No	No	No	T3	N0	No	No	61.42	63.95	No	No	No	No
M	54	Ex	Yes	O	No	No	G1	Neg	No	No	Yes	T2	N1	No	No	61.11	62.96	Yes	No	Yes	No
M	64	Ex	Yes	O	Yes	No	G3	Pos	Yes	No	Yes	T3	N2c	Yes	No	62.32	69.33	Yes	No	No	No
M	67	Ex	Yes	E	Yes	No	G3	Neg	No	No	No	T1	N2b	No	No	61.71	63.46	No	No	No	No
M	53	Ex	No	O	Yes	Yes	G3	Close	No	No	No	T3	N0	No	No	60.14	70.22	No	No	No	No
M	60	Yes	No	O	No	No	G2	Neg	No	Yes	Yes	T1	N3	No	No	68.96	71.67	No	No	No	No
F	62	Ex	No	O	Yes	No	G3	Neg	No	No	No	T2	N2c	Yes	No	46.95	66.19	No	No	No	No
M	57	Ex	No	O	Yes	No	G2	Pos	No	No	No	T3	N2c	No	No	61.06	68.28	No	No	No	No
M	59	Ex	Yes	O	No	Yes	G1	Neg	Yes	No	No	T3	N0	No	No	60.96	63.38	No	No	No	No
M	58	Ex	Yes	O	No	Yes	G2	Neg	No	No	No	T3	N2a	No	Yes	60.20	62.58	No	No	No	No
M	61	Yes	Yes	O	No	No	G2	Neg	No	No	Yes	T3	N1	Yes	No	37.90	61.76	No	No	No	No
F	71	Yes	Yes	O	No	No	G3	Close	No	No	No	T3	N0	No	Yes	58.40	68.12	Yes	No	No	No
F	62	Yes	Yes	O	No	Yes	G2	Neg	No	No	Yes	T3	N2c	Yes	Yes	59.71	63.74	Yes	No	No	No
M	60	Ex	Yes	O	No	Yes	G2	Neg	Yes	No	No	T4a	N0	No	No	60.33	63.04	No	No	No	No
M	41	Yes	Yes	O	No	Yes	G3	Neg	No	No	No	T3	N0	No	No	61.69	69.72	No	No	No	No
F	60	Ex	No	E	No	No	G3	Neg	No	No	No	T2	N2a	No	No	44.45	63.50	No	No	No	No
M	75	Yes	Yes	O	No	Yes	G2	Neg	Yes	No	Yes	T3	N2b	No	No	61.04	66.51	Yes	No	No	No
M	56	Ex	No	O	No	No	G3	Neg	No	No	No	T4a	N0	No	0	58.81	66.90	Yes	No	No	No

Abbreviation: E= endoscopic surgery, O= open surgery, Neg= negative, Pos= positive.

Comment to Supplementary Table S2: Of the six patients (35%) requiring the positioning of a temporary tracheostomy: two had acute respiratory distress, three severe laryngeal edema or stenosis requiring endoscopic dilatation, and one delayed tracheostoma healing. Tracheostomy was left in place in five patients for a median period of 23 days (IQR 19-90 days).

Both of the patients requiring temporary enteral nutrition had also respiratory distress requiring tracheostomy. One patient died (after 373 days) with both tracheostomy and percutaneous enteral gastrostomy still in place. For the second patient the feeding

tube was removed after one month but, due to the persistent swallowing defects, a plastic surgery at the base of tongue (lipofilling) was performed.

Table S3. Correlation between risk factors and long-term side effects.

	Temporary Tracheostomy due to Stenosis/Edema	Tracheostomy at Last Follow-up	PEG During Follow-up	Chondronecrosis	Enteral Nutrition
Sex (female vs male)	0.05	0.16	0.57	0.02	1
Radiation technique (VMAT vs 3D CRT)	0.30	0.64	0.69	0.12	1
Tracheostomy during radiotherapy (Yes vs no)	0.53	0.62	0.40	1	1
Surgery technique (Open vs endoscopic)	0.09	0.63	0.44	0.51	1
Enlarged tongue base (yes vs no)	0.57	1	0.53	1	1
Arytenoid removal (yes vs no)	0.71	0.09	1	1	0.26
Age	0.58	0.88	0.31	0.65	0.05

Table S4: Toxicity profile of patients treated with conservative surgery and postoperative radiotherapy for SG performed with no-IMRT techniques and current series.

	Treatment	Number of Patients	Permanent Gastrostomy	Persistent Aspiration	Permanent Tracheostomy	Acute Respiratory Distress	Laryngeal Stenosis	Laryngeal Necrosis
Steniger et al. 1997 [11]	3D CRT	17	35%	29.4%	23.5%	29%	NR	NR
Spriano et al. 2000 [10]	3D CRT*	56	0%	9%	1%	NR	NR	1%
Laccourreye et al. 2000 [24]	Co60	90	2.2%	3.3%	1.1%	NR	4.4%	5.5%
Garibaldi et al. 2009 [21]	2D/3D CRT	36	NR	NR	0%	3%	NR	NR
Current series	IMRT	20	0%	0%	5%	15%	15%	0%

* 25 patients were treated with non-standard (accelerated hyperfractionated) fractionation schedules.
